# The Dynamic Relationship between Dengue Virus and the Human Cutaneous Innate Immune Response

**DOI:** 10.3390/v16050727

**Published:** 2024-05-04

**Authors:** Michelle M. Martí, Priscila M. S. Castanha, Simon M. Barratt-Boyes

**Affiliations:** 1 Department of Infectious Diseases and Microbiology, University of Pittsburgh, Pittsburgh, PA 15260, USA; mmm265@pitt.edu (M.M.M.); pmd35@pitt.edu (P.M.S.C.); 2 Faculdade de Ciệncias Médicas, Universidade de Pernambuco, Recife 52171-011, Brazil

**Keywords:** dengue virus, innate immunity, cutaneous, human skin, skin explants, antiviral, interferon, dendritic cells, macrophages, keratinocytes

## Abstract

Dengue virus (DENV) is a continuing global threat that puts half of the world’s population at risk for infection. This mosquito-transmitted virus is endemic in over 100 countries. When a mosquito takes a bloodmeal, virus is deposited into the epidermal and dermal layers of human skin, infecting a variety of permissive cells, including keratinocytes, Langerhans cells, macrophages, dermal dendritic cells, fibroblasts, and mast cells. In response to infection, the skin deploys an array of defense mechanisms to inhibit viral replication and prevent dissemination. Antimicrobial peptides, pattern recognition receptors, and cytokines induce a signaling cascade to increase transcription and translation of pro-inflammatory and antiviral genes. Paradoxically, this inflammatory environment recruits skin-resident mononuclear cells that become infected and migrate out of the skin, spreading virus throughout the host. The details of the viral–host interactions in the cutaneous microenvironment remain unclear, partly due to the limited body of research focusing on DENV in human skin. This review will summarize the functional role of human skin, the cutaneous innate immune response to DENV, the contribution of the arthropod vector, and the models used to study DENV interactions in the cutaneous environment.

## 1. Introduction

Dengue is the most prevalent arboviral infection in the world [1,2,3,4]. There are currently four globally circulating serotypes of dengue virus, the causative agent (DENV1-4), that are related but serologically distinct [1,5]. This positive-sense single-stranded RNA (+ ssRNA) virus is a member of the *Flaviviridae* family and is primarily transmitted through the bites of female *Aedes aegypti* mosquitoes, and less commonly, *Aedes albopictus* [6,7]. DENV was endemic in only nine countries in 2009 but is now considered endemic in over 100, putting around half of the world’s population at risk [5,8,9]. This increase in endemicity is due to multiple factors including urbanization, global travel, increases in population density, and global warming causing geographic spread of the vector [1,3,5,10]. Predictive modeling has estimated that there are around 390 million global cases of DENV infection annually, with 96 million of these cases being symptomatic [11]. Although the mortality rate of DENV infections is not high, the disease substantially impacts the hospital systems and economic infrastructure of these endemic countries [12,13,14,15]. 

In 2009, the World Health Organization categorized the stages of disease as dengue with or without warning signs and severe dengue [16]. The disease begins with an acute febrile stage, where patients experience a combination of nausea, vomiting, aches, and lethargy [15,16,17,18]. This can be self-limiting or progress to severe disease in 2–5% of cases with plasma leakage, hemorrhage caused by severe thrombocytopenia, or organ failure [15,17,18,19]. The treatment for patients with severe dengue is limited to palliative care [18]. Although there are no approved therapeutics, recent in vivo and ex vivo work shows encouraging data for small-molecule inhibitors of DENV replication and mature virion formation [20,21,22,23]. Global efforts towards vaccine development have been complicated by the multiple serotypes of DENV and the risk of antibody-dependent enhancement, but several vaccine candidates are now approved or in the final stages of testing. Dengvaxia and Qdenga are tetravalent live-attenuated vaccines that are approved for use in select countries [24,25]. Dengvaxia requires a previous laboratory-confirmed infection prior to administration, while Qdenga does not. A promising third vaccine is the single-dose live attenuated Butantan-Dengue Vaccine, which is currently in phase 3 clinical trials [26].

Since DENV is transmitted by mosquitoes, the skin is the primary site of infection. The skin is the largest organ in the human body, covering the entire outer surface, and serves as the first barrier of defense against DENV and other arboviral infections [27]. As the female mosquito probes the skin in search of a blood meal, saliva containing vasodilating proteins and DENV is deposited into the epidermal and dermal layers [28,29,30]. Various permissive cells reside within these two layers including keratinocytes, Langerhans cells, macrophages, dermal dendritic cells, fibroblasts, mast cells, and endothelial cells [31,32,33,34,35,36]. This broad tropism allows ample opportunity for viral replication and spread. Other cells like neutrophils, natural killer cells, and innate lymphoid cells are not permissive or are not primary targets of DENV but do influence the early immune response to infection [30,37,38,39,40]. These early interactions in the skin remain understudied due to the paucity of available and translatable models to investigate the complex dynamic between the cutaneous environment and the virus. This review will cover the structure and function of human skin, the primary cutaneous innate responses to DENV, the role of the insect vector, and the evolution of models that have informed the field. 

## 2. Dengue Virion and Replication

The DENV genome is about 10 kilobases and translates into a polyprotein that is proteolytically cleaved into 10 proteins [41,42,43]. Encoded are three structural proteins: capsid (C), pre-membrane (prM), and envelope (E), and seven nonstructural (NS) proteins: NS1, NS2a, NS2b, NS3, NS4a, NS4b, and NS5 [41,42,43]. The NS proteins are necessary for viral replication but have also been implicated in the evasion of host immune responses [43]. Although the primary receptor for DENV is not known, heparin sulfate, mannose receptor (CD206), CD14, and dendritic cell-specific intercellular molecule-3-grabbing non-integrin (DC-SIGN) have all been implicated as receptors or co-receptors [44,45,46,47,48]. These are expressed on a variety of cell types present in the skin: keratinocytes, Langerhans cells (LCs), mast cells, fibroblasts, dermal dendritic cells (DCs), and macrophages [27,49]. When a mosquito deposits DENV into the skin, the E protein binds to one of the receptors and induces receptor-mediated endocytosis in a clathrin-dependent manner [41,43]. In the cell, endocytic acidification results in a conformational change in the E protein that allows fusion with the endosomal membrane and release of the genome into the cytoplasm [41,42]. The genome binds to the rough endoplasmic reticulum, where the polyprotein is translated and cleaved by a combination of viral and host proteases [42,43]. The NS proteins transcribe a (−) ssRNA complementary template from the (+) ssRNA genome that is used to produce more genomes for translation and virion production [43]. As more viral genomes and proteins are made, a genome combines with the E, C, and prM proteins to form an immature virion [42,43]. The prM protein is proteolytically cleaved in the Golgi network, resulting in a mature virion that buds out and infects neighboring skin cells [42,43]. Eventually, infected LCs and dermal DCs will migrate out of the skin into the lymphatic system and spread the virus to the lymph nodes, where secondary replication occurs [50].

## 3. The *Aedes aegypti* Mosquito as an Arboviral Vector 

The *Aedes aegypti* mosquito is the primary vector for DENV as well as yellow fever, chikungunya, and Zika viruses [51]. It is thought that these mosquitoes originated in sub-Saharan Africa, but with increased globalization, human movement, and climate change, *Aedes aegypti* have spread to more than 160 countries [51,52,53,54]. Global modeling studies have revealed that environmental changes have led to a global increase in suitability, which is projected to increase the number of mosquito generations in 2050 by 17% [53]. This emphasizes the need for research into therapeutics, vaccine development, and vector control for arboviruses, as they are an increasing threat to public health. This species has adapted to living in high-density population areas where stagnant water and bloodmeals for breeding are readily available [51,52,54]. The *Aedes aegypti* is equipped with a proboscis containing six stylets designed for feeding [55]. As it inserts the proboscis into the skin, the stylets penetrate and search for blood vessels while simultaneously releasing saliva, in a process known as probing [55]. To promote blood feeding, deposited saliva induces edema through histamine production and secretes proteins to increase blood flow. For example, sialokinin is a vasodilator, and FXa inhibitor prevents coagulation, both of which increase endothelial permeability and blood flow while simultaneously preventing blood clotting [56,57,58]. While the primary function of mosquito saliva is to facilitate bloodmeals, studies have revealed that it has immunomodulatory properties that inadvertently enhance infection of arboviruses in the skin. Sialokinin has been shown to influence cutaneous innate immunity by shifting from a T_H_1-mediated, antiviral cellular response to a T_H_2-mediated humoral response [59]. While it is thought that this evolved in humans to induce tissue repair and prevent infection by opportunistic extracellular pathogens, it does create an ideal environment for viruses to infect. Edematous skin retains virions deposited during probing, where permissive immune cells are recruited and antiviral responses are suppressed, resulting in enhanced infection [30]. 

## 4. The Structure and Function of Human Skin

The human skin is the largest organ of the human body and is the primary site of DENV transmission. The complete surface area accounts for approximately 15% of an individual’s body weight [27,49]. As the exterior barrier, the skin has many essential biological and physical functions. It not only serves as the first layer of protection against physical injuries, UV damage, and invading pathogens but is also responsible for sensation and the regulation of temperature and hydration through water retention and secretion [27,49,60,61]. The skin is comprised of two primary layers, the epidermis and dermis, connected by the epidermal–dermal junction. Although the structure of the skin is fundamentally similar across different parts of the human body, it is broken down into two major classifications. The first is the thick, hairless skin that covers the palms of the feet and hands, and the second is the thin, hairy skin that covers the rest of the body [27,49]. The primary difference between these layers is due to the functionality of the parts of the body they cover. The palms of the feet and hands require more strength and durability for the physical demand they incur; therefore, they have an extra layer, the stratum lucidum, and higher levels of keratinization in the epidermis, as well as lower sensory sensitivity. The skin everywhere else undergoes less mechanical stress and therefore does not have the extra layer and has less keratinization in the epidermis as well as higher sensory sensitivity [27,49,60,61]. It has been shown that mosquitoes are more likely to bite exposed parts of the arms, legs, and abdomen [62]. Since DENV is spread through a mosquito bite, this review will focus on the thin, hairy skin that covers the arms, legs, and abdomen.

### 4.1. Epidermis

The epidermal layer is the outermost layer exposed to the environment. It is a self-regenerating layer of stratified epithelium. The primary cell type is the keratinocyte, which makes up 90–95% of the cells [27,49]. LCs, Merkel cells, melanocytes, and a few lymphocytes make up the remaining 5–10% [27,49]. Each of these cells has its unique role and function in maintaining skin homeostasis, response to pathogens, and tissue repair [60].

The epidermis is broken down into four sublayers. The first is the stratum basale, which is a single layer of keratin (K) 5-, K14-, and K15-expressing cuboidal keratinocyte stem cells that are continuously undergoing mitosis and differentiation into transient progeny that replenish the epidermis [27,49,60]. After a cell completes mitosis, the daughter cell moves suprabasally, continuously undergoing structural and biochemical changes, including changes in keratin expression, an increase in keratinization, flattening of its shape into a squamous cell, pyknosis, and loss of the nucleus [27,49]. Ultimately, they become terminally differentiated corneocytes and desquamate, or flake off. This process takes around 52–75 days to completely replace and replenish the layer in humans [49]. This is also the sublayer attached to the basement membrane that connects the epidermis to the dermis via the epidermal–dermal junction. Moving suprabasally, the layers that follow are the stratum spinosum, the stratum granulosum, and the stratum corneum. The stratum spinosum is characterized by K1- and K10-expressing mature keratinocytes that have differentiated and moved up from the stratum basale [27,49,60]. Next is the stratum granulosum, which consists of keratinocytes that have continued producing keratin, primarily K2E, and begin to flatten in shape [27,49,60]. Finally, the stratum corneum contains only corneocytes, which are terminally differentiated keratinocytes that have undergone pyknosis and are devoid of any nucleus [27,49]. These are the cells that will eventually desquamate as the skin replenishes itself. LCs are found through all of the sublayers but are predominately found in the stratum spinosum. Merkel cells and melanocytes, on the other hand, are only found in the stratum basale. 

Since the epidermis is a highly mitotic area that is constantly replenishing itself, it creates the perfect environment for viral replication. DENV takes advantage of host transcription and translation proteins to replicate its virions, and these are the most active in dividing cells [41,43]. In the epidermis, the stratum basale is the most actively replicating layer, which would explain why researchers have seen infection of basal keratinocytes in intact human skin [31,63]. Keratinocytes are known to be immune sentinels that release cytokines to alert the body of an infection, induce an innate immune response, and recruit cells to the site of infection [64,65,66]. However, the exact understanding of what specific keratinocytes are infected and their contribution to the spread of DENV in the epidermis has yet to be entirely understood. 

### 4.2. Dermis

The dermis is the second layer of human skin that lies beneath the epidermis and is responsible for the strength, resilience, and flexibility of skin. Its thickness ranges from 2 mm to 4 mm, depending on the location of the body [27]. The dermis is significantly less cell-dense compared to the epidermis but has higher cellular diversity. Dermal cells are categorized in one of two ways: the migrant and the permanent [49]. Migrant cells are those that originate in the bone marrow, travel through the blood to the dermis, and take residence. This primarily includes dermal DCs, macrophages, mast cells, eosinophils, T cells, and B cells [49]. These are all immune cells involved in recognition, recruitment, and response to invading pathogens. Permanent cells are those that make up the framework and structure [49]. This includes fibroblasts, the nerves that innervate the dermis, and the blood vessels that provide oxygen and nutrients. Fibroblasts make up the majority of dermal cells and are responsible for synthesizing the components of the extracellular matrix [49]. This matrix is comprised of ground substance and a variety of collagen and elastin fibers. The ground substance is an amorphous gel containing proteoglycans, glycosaminoglycans, and water that is present in most types of connective tissue [67]. It provides a matrix for cellular movement, intercellular attachment, and dermal homeostasis through water and electrolyte balance [67]. The collagen in adult dermis is made up of 80–85% Type I and 15–20% Type III [27,49,67]. Type I collagen is coarse and densely packed with fibers to provide strength and structure, whereas Type III has less densely interwoven fibers that form a fine, thin collagen [27,49,67]. 

The dermis has two sublayers, the papillary and the reticular layers, making up 20% and 80% of the dermis, respectively [49,61]. The papillary layer is directly below the epidermis. It is named after the hallmark papillae, or rete ridges, that are found interdigitating the epidermis. This layer provides physical structure and nutrient exchange to the epidermis [27,49,61]. Type III collagen bundles make up most of the collagen present, with a few elastin fibers woven in. The reticular layer provides the majority of the skin’s mechanical strength and elasticity [27,49,61]. This is due to the thick interwoven bundles of Type I collagen and interspersed elastin fibers that are found here. Together, the epidermal and dermal layers are a resilient and robust tissue that can dynamically respond to a myriad of stressors that the human body encounters. 

Although the dermis and epidermis are two physiologically and functionally different layers, their response to viral infections is a coordinated effort. When a DENV-infected mosquito probes the skin, it releases virus into both layers, where nearly every cell type has been shown to be permissive to infection. It is not clearly understood which target cells are infected first, but it is known that all these cells are involved in inducing an innate response to DENV. Infection of either layer results in interlayer communication to alert and recruit immune cells to contain viral replication and prevent the systemic spread of the virus to lymphoid tissue. There is a lot of information on how this coordinated response is carried out, yet the exact mechanisms that are active in the cutaneous environment in response to DENV are not fully defined. 

## 5. Immune Responses to Viral Infections in the Skin

The skin has a complex repertoire of immune defenses to combat the extensive array of pathogens it encounters. This requires the cutaneous innate immune response to have the capability to broadly respond to and simultaneously communicate and recruit the adaptive response to any invading microbe. For DENV, there are three primary classes of innate immunity in the skin, which will be discussed here: antimicrobial peptides, pathogen recognition receptors, and cytokines. 

### 5.1. Antimicrobial Peptides 

The first key component of cutaneous antiviral defense is a class of small peptides known as antimicrobial peptides (AMPs). There are two main categories of AMPs, including cathelicidins, like LL-37, and defensins α/β [60,68,69,70]. Under normal conditions, human skin constitutively produces AMPs, but when stimulation from tissue damage or pathogen invasion occurs, that production significantly increases. This balance between constitutive and increased production is imperative, as producing too much or too little is associated with various skin conditions like rosacea or psoriasis. LL-37 is a well-categorized AMP that has antibacterial, antiviral, and alarmin activity. It is a 37-amino acid peptide that produces an α-helix that can directly disrupt the viral envelope or the bacterial membrane, resulting in a detergent-like effect [68,69]. When DENV is pretreated or simultaneously exposed to LL-37, it significantly reduces infection and viral RNA genome copies in macrophages and Vero cells [71,72]. LL-37 also has alarmin activity, as it can interact with various receptors, including G protein-coupled and toll-like receptors, to induce downstream inflammatory signaling [68]. This results in the production of cytokines like IL-6, IL-10, IL-8, and IL-18, and chemokines to increase cell migration to the site of infection and activate tissue-resident cells to stimulate innate responses [68]. 

### 5.2. Pattern Recognition Receptors

**Figure 1:**
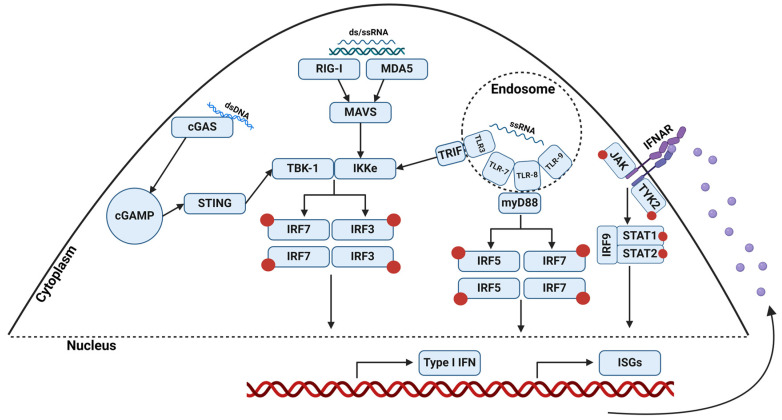
Schematic of pattern recognition receptors (PRRs) involved in intracellular viral sensing and downstream signaling. TLR-3/7/8/9, RIG-I, and MDA5 are ds- or ssRNA-sensing proteins, whereas cGAS is a dsDNA-sensing protein. These PRRs culminate in the recruitment and phosphorylation of interferon regulatory factors (IRFs). Activated IRFs translocate to the nucleus and bind IFN-stimulated response elements (ISRE) that induce transcription of type I interferons (IFNs) and select IFN-stimulated genes (ISGs). Released type I IFN binds to IFN-αβ receptors (IFNAR) and induces the JAK-STAT signaling pathway. JAK-Tyk2 kinases dimerize, and the activated form phosphorylates signal transducers and activators of transcription 1 and 2 (STAT). IRF9 is recruited to this complex and nuclearly translocates, inducing the expression of ISGs. These pathways create a positive feedback loop that amplifies the transcription of IFNs and ISGs in the skin, fostering an antiviral environment to combat viral infections. Red circles represent phosphorylation.

Pathogen recognition receptors (PRRs) are another vital defense mechanism in the skin (Figure 1). PRRs are genetically encoded receptors that recognize pathogen-associated molecular patterns and initiate a cascade of signaling events to activate antiviral immune responses. Although the skin expresses nearly every PRR, for DENV infections, toll-like receptors (TLRs), RIG-I-like receptors, and cyclic-GMP-AMP synthase (cGAS) are the most crucial [73,74]. Signaling through these sensors leads to the recruitment of transcription factors, known as interferon regulatory factors (IRFs), that can bind to interferon-stimulated response elements and induce transcription of type I interferons (IFNs) and some IFN-stimulated genes (ISGs) [73,74,75]. In humans, there have been upwards of 1000 ISGs discovered through microarray studies [76]. This includes a wide range of antiviral proteins, including OAS1/2/3, RNase L, Type 1 IFNs, and viperin, to name a few. 2′-5′-oligoadenylate synthase is a family of antiviral enzymes that restrict viral replication through the activation of RNase L, a ribonuclease that digests dsRNA [77]. Viperin, on the other hand, is hypothesized to interact with the NS3 protein to restrict viral replication, although the exact mechanism is unknown [78]. 

Signaling through PRRs acts as a positive feedback loop; as more type I IFNs are produced, signaling is amplified. TLRs are a family of transmembrane proteins that recognize common motifs in the viral genome that initiate signaling. TLR 3, 7, 8, and 9 are viral dsRNA- or ssRNA-recognizing endosomal receptors [73,74,75]. Upon recognition of dsRNA, TLR 3′s adaptor protein, TIR-domain-containing adaptor-inducing IFN-β, recruits IRF 3 and 7 [73,74,75]. When TLR 7/8/9 recognizes ssRNA, their adaptor protein, myeloid differentiation primary response 88 (myD88), recruits IRF 5 and 7 [73,74,75]. RIG-1-like receptors like retinoic acid-inducible gene-I (RIG-I) and melanoma differentiation factor-5 (MDA5) are cytosolic RNA sensors that also recruit IRF 3 and 7 [73,74,75]. Curiously, cGAS is a DNA-specific cytosolic sensor, and DENV does not have a DNA intermediate. Upon DNA stimulation, cGAS converts ATP and GTP to cyclic GMP-AMP, which activates the stimulator of interferon genes (STING) [79]. STING converges at the same pathway as RIG-I and MDA5, ultimately resulting in IRF3 recruitment and ISG production. It was later discovered that upon DENV infection, a source of endogenous mitochondrial DNA is released, and this acts as the DNA source for activation of the cGAS-STING pathway [79]. This indirect RNA virus sensing and activation of IFNs and ISGs further amplifies the innate response and prevents viral evasion of the immune response through alternative activation pathways. The induction of PRR signaling induces the production of type I IFN signaling through the JAK-STAT pathway and eventually ISG production. The JAK-STAT pathway will be discussed in the section that follows. 

### 5.3. Cytokines

The final piece of the cutaneous defense system is the soluble proteins that mediate cell-to-cell communication. The term cytokine encompasses a large family of proteins that can be secreted from cells to modulate autocrine or paracrine immune signaling. This includes interleukins, chemokines, and IFNs. Interleukins modulate the inflammatory response, chemokines recruit immune cells to the site of infection, and IFNs induce an antiviral response. The balance between these three is crucial, as dysregulated responses can result in disease [80]. Overstimulation of cytokines early in the response to DENV, known as cytokine storm, is a hallmark of severe disease [80]. This overactive systemic immune response overwhelms the immune system and results in immune-mediated pathology, further emphasizing the importance of a balanced response. 

Interleukin and chemokine release is primarily associated with the induction of a pro-inflammatory response. This includes activation of endothelial cells and increased expression of cellular adhesion molecules like intercellular adhesion molecule-1 (ICAM-1) and E-selectin [81]. This modification of vasculature tight junctions allows chemokines, like CCL2 and CXCL8, to attract lymphocytes, monocytes, and neutrophils in a gradient-dependent manner to the skin [82,83]. Tumor necrosis factor-α (TNF-α) is one of the cytokines involved in vascular activation that has also been implicated in the immunopathology of vascular leakage in severe dengue [84,85]. It is highly expressed in the skin and is known as a master regulator for skin diseases, wound healing, and pathogen response [85]. It results in downstream activation of nuclear factor kappa-light-chain enhancer of activated B cells (NF-κβ), which increases the expression and production of inflammatory cytokines, i.e., IL-18, IL-1α, and IL-1β [85]. Many cytokines activate the NF-κβ signaling pathway, resulting in positive feedback for the production of pro-inflammatory cytokines and adhesion molecules. In short, the binding of interleukins to their cognate receptors results in the activation of kinase cascades that eventually leads to phosphorylation and degradation of the NF-κβ regulator, IκB [86,87]. Freed NF-κβ nuclearly translocates and initiates transcription of a variety of pro-inflammatory proteins. IL-1′s, IL-6, IL-18, and IL-37 are all highly expressed in the skin and have also been associated with increased inflammation and dengue disease severity [19,87,88,89].

IFNs are arguably the most critical cytokine in cutaneous viral infections, as they induce and amplify the transcription of IFN-stimulated genes (ISGs). There are three major types of IFNs: Type I (α/β), Type II (γ), and Type III (λ). Type II IFN is produced predominantly by T-cells and is considered an adaptive response cytokine. Types I and III are produced in response to viral infections, with the primary goal of stopping viral replication and limiting the spread of infection. Both Type I and III interferons signal through the JAK-STAT signaling pathway. The binding of Type I and III IFN to their respective transmembrane receptors results in the dimerization of cytoplasmic Janus Kinase (JAK) and Tyrosine Kinase (TYK2) [90,91,92]. Dimerization leads to activation via cross-phosphorylation of the JAK and TYK2 kinases, which then phosphorylate the signal transducer and activator of transcription (STAT) 1 and 2 [90,91]. IRF9 is recruited to this STAT1/2 complex, creating IFN-stimulated gene factor 3 (ISGF3) [90,91]. ISGF3 is functionally comparable to IRF3, in that it is translocated to the nucleus and binds to IFN-stimulated response elements to induce ISG expression [90,91]. Akin to PRR signaling, IFN creates a positive feedback loop, inducing more ISG production to restrict viral replication. In conjunction, AMPs, PRRs, and cytokines provide a significant barrier that the virus must evade upon entry into the skin. Reducing viral replication at the site of transmission prevents the virus from spreading systemically, making the dynamic between DENV and the cutaneous environment a vital area of research. 

## 6. Models to Study Cutaneous DENV Infections 

The current understanding of the virology, host immune response, and immunopathology of DENV is derived from a combination of laboratory systems and models. When it comes to the interaction between the virus and the skin’s microenvironment specifically, information is limited. Using primary human cutaneous cells, small and large animal models, and ex vivo human skin explants, the early interactions at the site of transmission have begun to be teased apart. The following will cover the evolution of these methods and how they have influenced the field of knowledge. 

### 6.1. Primary Human Skin Cells

Primary human cutaneous cells can either be isolated from intact tissue discarded from elective cosmetic surgeries such as abdominoplasties and panniculectomies, from biopsy punches taken from human volunteers, or circumcisions. These cells are an invaluable tool for studying the viral–host interactions and the permissiveness of specific tissue-resident immune and nonimmune cells to DENV. The following section will go into detail on some of the pivotal studies that use primary cells to investigate the susceptibility of keratinocytes, mononuclear phagocytes, fibroblasts, and mast cells to DENV and their subsequent innate immune responses. A summary of the studies, specifically using human skin, is shown in Table 1.

#### 6.1.1. Keratinocytes 

DENV has been shown not to be limited to one receptor or one cell type, making it an elusive and challenging topic to study. This has led researchers to question what cell types are permissive to DENV. Keratinocytes make up a large proportion of the cells in the skin, but their susceptibility to DENV and their role as immune cells have been questioned. 

One of the first breakthroughs in understanding the susceptibility of keratinocytes was in 2011. Primary keratinocytes isolated from neonatal foreskins were infected with DENV-2 and shown to be permissive to DENV infection by quantifying the number of viral genomic copies [64]. This work highlighted the importance of keratinocytes as immune sensors. Isolated keratinocytes increased transcription of PRR’s RIG-I, MDA5, and TLR-3, in addition to transcription of ISGs like Type I and III IFNs, AMPs, and PRR adaptor proteins [64]. This confirmed, for the first time, the permissiveness of keratinocytes and their role in establishing an antiviral environment in response to infection. This established a model for infecting primary human keratinocytes with DENV. This work was followed up by introducing a third variable into the system: Aedes aegypti salivary gland extract (SGE) [66]. The ability to investigate the role of saliva during infection is crucial, as saliva itself has immune-modulating properties that can enhance infection [28,29]. By infecting cells in the presence or absence of SGE, it was demonstrated that DENV infection of keratinocytes was enhanced when SGE was present [66]. Viral RNA copies and transcription of AMPs significantly increased at 6 and 24 hours post-infection (hpi) [66]. Interestingly, the early type I IFN response seemed to be less affected by the presence of SGE, since there were no significant differences at 6 hpi, but they were significantly increased at 24 hpi. This work not only established a model to study DENV infection of primary keratinocytes but also demonstrated the effect that the presence or absence of saliva has on infection. 

Another model was developed using HaCaT cells, an immortalized keratinocyte cell line. Cultured HaCat cells were infected with DENV-2 and analyzed for susceptibility to infection and subsequent innate responses [69]. These data confirmed that even an immortalized keratinocyte cell line is subject to replicative and productive DENV infection by immunofluorescent staining of NS3 and NS5 [69]. This also confirmed the importance of keratinocytes in inducing an innate immune response. Infection led to an increased transcription of PRRs TLR-3 and RIG-I [69]. Increased recognition and signaling through PRRs resulted in higher levels of type I and III interferons and the secretion of IL-6, IL-8, and TNF-α at 24 hpi [69].

#### 6.1.2. Mononuclear Phagocytes

In healthy human skin, there is a diverse group of tissue-resident mononuclear phagocytes (MNPs) that screen the cutaneous environment for foreign antigens. The epidermis is home to LCs, and the dermis is home to multiple DC subsets (CD1c+, CD141+, and CD14+), macrophages, and a few LCs [101]. CD1c+ DCs prime T cells, CD141+ DCs can cross-present antigen, and CD14+ DCs are functionally more similar to monocytes [33,101,102]. Much of the work investigating DENV infection of MNPs has been accomplished using monocytes isolated from blood samples that are differentiated into DCs and macrophages in vitro [103,104,105,106]. Although this is a good proxy for studying MNP responses to DENV, it does not fully recapitulate skin-resident MNP responses to DENV at the site of transmission. In the last 25 years, there have been many advances in the understanding of cutaneous MNP function during DENV infection using primary cutaneous cells. Using discarded intact tissue from elective surgeries that has been enzymatically digested, primary dermal single-cell suspensions can be collected and infected in vitro. When dermal cells are exposed to DENV, infection of CD1c+, CD141+, and CD14+ DCs, LCs, and macrophages is seen at 48 hpi [33]. Dermal CD14+ DCs and LCs were consistently infected at higher rates, regardless of serotype [33]. This work also revealed that the infection of all MNPs could occur in a DC-SIGN-independent manner, as anti-DC-SIGN blocking antibodies had no effect on infectibility [33]. This further corroborates that DENV tropism is not specific to one cell type or one receptor. The permissiveness of DC subsets, LCs, and macrophages to DENV was later validated and confirmed [93]. This was taken a step further by analyzing the indirect effect of mosquito saliva on infection of MNPs. When a mosquito releases saliva into the skin, it promotes the activation and degranulation of mast cells, eosinophils, and basophils. This causes an influx of IL-4 production in the dermal layer. Schaeffer et al. showed that the presence of IL-4 enhanced infection of DCs and macrophages, providing further evidence for the importance of mosquito saliva in enhancing infection [93]. In another study, skin biopsies were taken from patients within 24 h of dengue shock syndrome onset and digested into single-cell suspensions [94]. Decreased levels of CD1a+ DCs and increased levels of T cell activation were observed in the skin [94]. Although these samples were collected much later through the course of the infection, it emphasizes the benefit that tissue samples at later time points from patients can provide. 

In resting, non-inflamed skin, dermal DCs and LCs are constantly patrolling the tissue for foreign antigens [107]. Upon recognition of a pathogen, DCs undergo maturation and expression of proteins for homing to lymphoid tissue to activate lymphocytes [107]. The susceptibility of immature versus mature DCs to DENV has been an ongoing question. Infecting mature LCs that migrated out of the tissue from cadaveric skin in vitro resulted in only 2% of infection [32]. These data confirmed in physiologically relevant cells what the group had previously seen when infecting immature and mature monocyte derived DCs isolated from blood samples [32]. It was later verified in a study that directly compared primary immature and mature skin-derived LCs. Using discarded skin, Helgers et al. used two different methods to isolate immature and mature LCs. To isolate immature LCs, the tissue was enzymatically digested, and LCs were positively selected using a magnetic column. On the other hand, mature LCs were collected from tissue that was left in culture for 3 days, allowing mature LCs to migrate out into the media [95]. Confirmation of the LC state was performed by measuring the expression of CD80, CD83, CD86, and langerin [95]. Immature LCs express low levels of CD80, CD83, and CD86 but high levels of langerin, whereas mature LCs express the opposite phenotype. The data suggest that both LC phenotypes are permissive to infection ex vivo, but their susceptibilities differ. Immature LCs are permissive to infection at a low MOI, whereas mature LCs require more of the virus for productive infection [95]. 

#### 6.1.3. Fibroblasts

Fibroblasts are one of the primary cell types found in the dermal layer. Similar to keratinocytes in the epidermis, they act as immune sentinels in the dermal layer. They primarily aid in cell-to-cell communication and recruitment of other immune cells to the site of infection [34]. As early as in 1992, primary skin fibroblasts isolated from skin biopsies were shown to be infectable by DENV-2 as early as 4 hpi and reached maximum levels at 24 hpi [34]. These data also revealed that dermal fibroblasts produce various cytokines to help initiate an immune response in the dermal layer, mainly IFN-β, IL-6, and GM-CSF [34]. Granulocyte macrophage-colony stimulating factor is a growth factor that stimulates the maturation and differentiation of monocytes and macrophages [108]. This points to the importance of fibroblasts in paracrine signaling to activate dermal immune cells in response to DENV. Another common source of primary fibroblasts is neonatal foreskins. Using these primary cells, it was confirmed in a different model that fibroblasts are permissive to DENV [96]. 

Although these data showed the susceptibility of fibroblasts to DENV and began to hint at their role in the skin’s response to infection, it was still unclear what their exact role was in fostering the antiviral environment. Using healthy skin, primary human fibroblasts were isolated to further elucidate their immune response to DENV [97]. The data showed upregulation of TLR-3 peaking at 12 hpi and RIG-I peaking at 36 hpi [97]. Looking further into the signaling pathways, these authors showed that DENV induces nuclear translocation of IRF3, which would result in the transcription of ISGs [97]. Aside from signaling pathways, there was also evidence of DENV-mediated induction of TNF-α, IFN-β, and AMPs [97]. Using a normal fibroblast cell line, WS1, this signaling was further investigated. This confirmed the upregulation of RIG-I, IRF-3, TNF-α, and IFN-β seen in primary cells, but also revealed upregulation of IL-1β, IL-6, IL-8, IFN-α, IFN-λ, CXCL10, and IRF7 [98,99]. Looking downstream of PRR recognition, transcription factors IRF3 and NF-κβ must be phosphorylated to translocate to the nucleus and induce transcription [98,99]. Phosphorylated IRF3 and NF-κβ were found in DENV-infected fibroblasts, revealing their ability to induce downstream signaling of PRRs and contribute to the dermal antiviral response [98,99]. 

#### 6.1.4. Mast Cells

Mast cells are a type of granulocyte present in the dermal layer of the skin. In the cytoplasm of these cells, there are granules containing histamine and other chemical modifiers that can be released upon stimulation [109]. This process of degranulation induces endothelial cell activation and vascular permeability, which have both been linked to disease pathology [109,110]. There has been extensive work using cell lines and mast cells derived from human cord blood that has shown FcγRII-dependent enhancement of infection, upregulation of CCL3, CCL4, CCL5, IL-1β, and IL-6, and TNF-α-mediated endothelial activation [111,112,113,114,115]. There is limited work using primary mast cells isolated from human skin. Using discarded intact surgical tissue, isolated mast cells were shown to be readily infected with DENV in the absence of antibody-dependent enhancement [35]. Infection-induced upregulation of CCL5, IL-6, and IL-8 and increased activation of co-cultured endothelial cells, confirming data collected from cell lines and cord blood-derived mast cells [35]. This further emphasized the impact of mast cells during not just secondary but also primary infections in human skin. It was previously shown in mice that when mast cells degranulate, extracellular granules are released that travel to lymphoid tissue to deliver chemical immune signals [116]. Whether these granules were infectious or not during the infection was unknown. By isolating and infecting primary cutaneous mast cells with DENV, it was revealed that cytoplasmic and extracellular granules colocalized with DENV that were still infectious. This led to the hypothesis that extracellular granules are capable of disseminating virus to the draining lymph nodes [35].

### 6.2. Small and Large Animal Models 

Although primary cells have provided insight into the susceptibility of various cell types in the skin and the innate responses they induce, this system is limited in the information it can provide. In culture, cells are analyzed in isolation from each other rather than within the framework of the tissue. Skin is a complex organ with a diverse range of cell types, and intercellular communication upon DENV infection is a critical component to understanding cutaneous DENV infections. Small and large animal models, like mice and nonhuman primates, are frequently used in laboratory research but are primarily used to study the systemic spread of DENV infection, clinical presentation, therapeutic development, and vaccine development [117,118,119,120,121,122,123]. 

One of the primary reasons for limited animal model research of the cutaneous environment is due to differences in viral evasion of the innate response. In humans, the DENV NS5 protein can bind and target STAT2 for degradation [75,124,125]. DENV NS2B3, a complex of NS2b and NS3, has been shown to proteolytically cleave human STING and cGAS [126,127,128,129]. In combination, this inhibits the human antiviral response by antagonizing the signaling pathways to produce ISGs, IFNs, and cytokines [128]. In mice, DENV NS5 cannot bind to STAT2, and in mice and nonhuman primates, NS2B3 cannot cleave STING or cGAS [126,130,131]. This prevents evasion of the innate response in both animals. This explains why immunocompetent mice are considered resistant to DENV infections and must be inoculated with high doses, use mouse-adapted strains, or infect transgenic mice to see disease comparable to humans [117,122,123,132,133,134,135]. Even though DENV exists in a sylvatic cycle with nonhuman primates, they do not produce high viremia or present with clinical manifestations, despite producing antibodies to the virus [136,137]. In a laboratory setting, rhesus macaques, bonnet macaques, marmosets, and many other nonhuman primate species have been tested to model DENV infections, but all require high-dose inoculations [136,137]. 

One of the significant limitations of primary skin cells and skin explants is the lack of vasculature, making it challenging to look at the recruitment of cells to the skin or the migration of cells from the skin to draining lymph nodes. Mice that lack Type I IFN receptors (IFNAR −/−) were used to highlight the role of DCs in activating the adaptive response while inadvertently disseminating DENV in lymph nodes [33]. They also showed a significant increase in the recruitment of circulating monocytes into the skin at the site of inoculation [33]. The same phenomenon was seen in a model of DENV infection in cynomolgus macaques [94]. Macaques were inoculated with DENV-2 in the thigh, and at 4 dpi, a biopsy was taken from the inoculated and contralateral thighs for analysis [94]. A significant infiltration of monocytes was detected, although direct infection of monocytes was not detected [94].

A mouse model using wild-type C57BL/6 and mast cell-deficient mice demonstrated the essential role of mast cells in reducing viral burden through the recruitment of natural killer cells to the site of infection [39]. This model also revealed that murine mast cells sense and respond to viruses through MDA5 and RIG-I, resulting in the production of IFN-α and TNF-α [39]. These data suggest that mast cells play an essential role as immune cells in the skin. Using discarded dermal tissue from cynomolgus macaque necropsies, DENV infection of primate mast cells was also confirmed [39]. Another mouse model was used to answer the earlier hypothesis of whether extracellular granules can disseminate virus to the lymph nodes. Extracellular granules isolated from infected human mast cells were injected into the footpads of AGB6 mice, and viral infection was quantified at 24 hpi [35]. Half of the mice infected with these granules had DENV+ infection in the draining lymph nodes, providing evidence of an alternate viral dissemination pathway [35]. 

### 6.3. Ex Vivo Human Skin Explants

**Figure 2:**
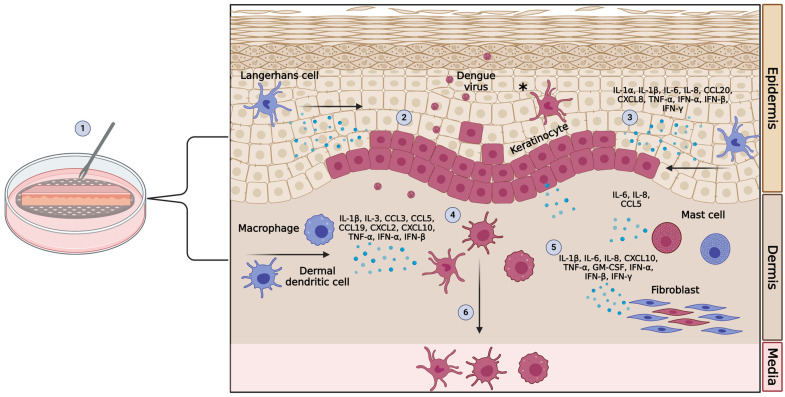
Schematic of an ex vivo human skin explant model. (1). Dengue virus (DENV) is inoculated into tissue with a bifurcated needle. (2). DENV infects keratinocytes and nearby Langerhans cells. (3). Infected and bystander keratinocytes produce cytokines and chemokines to activate the immune response and recruit immune cells from surrounding tissue to the site of infection. (4). The virus spreads to the dermis and infects fibroblasts, mast cells, dermal dendritic cells, and macrophages. (5). Infected and bystander dermal cells produce cytokines and chemokines to recruit cells and prevent the spread of infection. (6). Infected Langerhans cells and dermal dendritic cells migrate out of the tissue into the media, mimicking the migration to lymphoid tissue that occurs in humans. * Red = infected cells, blue = uninfected cells. Cells not drawn to scale; lymphocytes and natural killer cells not pictured.

The issue with cell culture and animal models is that neither system fully recapitulates the complexity of the cutaneous environment. In contrast, in ex vivo human skin explant models, tissue from cadavers or elective cosmetic surgeries is left intact and placed in culture at the air–liquid interface (Figure 2). By keeping the tissue viable and in its original structure, it provides an excellent model for studying the cutaneous environment. This also retains the original framework, maintaining the cellular communication between the epidermal and dermal layers. The skin microenvironment is a complex system that can dictate whether infection will be contained or whether it will lead to dissemination and cause clinical manifestations. For this reason, explant models have become indispensable for investigating viral–host interactions at the site of transmission. 

Over the last 25 years, these models have become more widely used and the techniques for inoculating tissue have changed (Table 1). One of the first explant models involved taking full-thickness cadaveric tissue and injecting DENV with a 27-gauge needle into the epidermal and dermal layers [32]. The tissue was left in culture for 48–72 h, allowing DCs to migrate into the media and the percentage of infected cells to be quantified. When skin was infected ex vivo, around 60–80% of migrated dermal DCs and LCs were positive for DENV [32]. These authors showed that mature DCs were not permissive to infection, but immature DCs were highly permissive [32]. As discussed in the primary cell section, this was later confirmed in studies using primary, immature, and mature LCs infected in vitro [95]. Infectivity of immature vs. mature DCs was also shown in an explant model where the epidermis was separated from the dermis and then infected [95]. This work demonstrated that of the LCs that migrated from the epidermis, those lacking langerin were infected but LCs expressing langerin were not, suggesting that mature, langerin-deficient LCs are not targets of DENV [95]. Further validation using a biopsy punch from a human volunteer 12 days after receiving a live-attenuated tetravalent DENV vaccine was administered. Histological staining revealed DENV+ DCs and LCs, providing more evidence of their infectability in humans; however, they were unable to distinguish immature from mature DCs in this study [32,103]. 

Aside from cadaveric skin, another source of intact human tissue is elective cosmetic surgeries. This discarded, healthy tissue is commonly used for the isolation of primary cutaneous cells and explant models. The collection and processing of the tissue across labs is consistent; however, the method of infection can vary. One example is scarification. Using a 30-gauge insulin needle, DENV is deposited on the surface and is gently scraped into the tissue. After 2 hpi, the excess virus is removed, and the explant is placed in culture for up to 120 h [63]. Data using this methodology revealed limited infection in the basal layer of the epidermis and no infection in the dermal layer [63]. Despite this, the dermal layer showed increased activation of dermal DCs with increased expression of CD80 and CD83 [63].

Explants from elective procedures have also been inoculated using mosquitoes infected with DENV-2. At 24 hpi with Aedes aegypti, DENV+ mast cells were detected by immunofluorescent staining and quantification of viral genomic copies [35]. This system, using the natural route of infection, revealed that mast cells are an early target of DENV infection in intact human skin. 

Using non-cadaveric tissue, one model mimics the natural route of infection using an alternative inoculation technique. DENV-2 was inoculated by stabbing the virus into the skin with a bifurcated needle [31]. By stabbing the virus into the skin, it is intended to mimic mosquitoes probing during the natural transmission process. Similar to other models, at 2 hpi, excess virus is removed, and the tissue is transferred to a piece of mesh, creating an air–liquid interface [31]. It remains in the incubator for up to 24 hpi [31]. This technique provides samples of cells at the site of infection, cells that locally migrate to the site from surrounding tissue, and cells that emigrate into the media (Figure 2). Immunofluorescent staining of tissue revealed infection of nearly every cell type in the skin: keratinocytes, LCs, macrophages, dermal DCs, fibroblasts, and mast cells. The earliest virus replication, at 6 hpi, was seen in basal keratinocytes, and by 48 hpi, keratinocytes made up around 60% of all infected cells. The remaining 40% comprised LCs, macrophages, dermal DCs, fibroblasts, and mast cells [31]. This confirmed what had been seen and contributed more evidence to the theory of keratinocytes being a primary target of DENV in the skin [32,35,64,66,97]. In the presence of virus, LCs and dermal DCs emigrated to the media, and the expression of IL-1α, IL-1β, IFN-α, and CCL20 was upregulated [31]. Immunohistochemistry revealed that infected keratinocytes were the primary producers of IL-1β and CCL20. In addition to looking at the effects of DENV, microneedle arrays were used to manipulate the cutaneous immune environment to identify the mechanistic role of upregulated cytokines. Arrays loaded with neutralizing antibodies to IL-1α, IL-1β, CCL20, CXCL8, or isotype controls were applied to tissue immediately after DENV infection. Strikingly, by blocking IL-1β, total infection in the epidermis decreased by 33% and in the dermis by 65% [31]. Interestingly, neutralizing IL-1β only reduced infection of LCs and not keratinocytes in the epidermis [31]. These data showed that the inflammatory response of the skin to DENV infection itself mediates local migration of skin-resident LCs, DCs, and macrophages, which then become infected and spread the virus throughout the body (Figure 2). Increased levels of IL-1β have been clinically associated with severe disease [19,80,88], and the data in the skin explant model suggest that IL-1β enhances infection right at the time of initial virus transmission in the skin. 

While explant models have proven to be an indispensable tool for studying DENV in the skin, some limitations must be considered. Explants are ex vivo pieces of tissue that do not have any associated vascularity. Because of this, the role of innate immune cells that migrate to the skin in response to infection cannot be studied. Neutrophils are not present in healthy skin but are highly recruited from the vasculature upon tissue damage and saliva deposit from a mosquito bite [30]. Natural killer cells are present in small numbers but are also recruited from circulation in response to infection and help prevent systemic spread [40]. Another limitation is the method of inoculation. Needle inoculation or scarification is frequently used for convenience, but to fully recapitulate natural transmission DENV-infected Aedes aegypti mosquitoes should be used to inoculate human skin [35]. Co-inoculation of skin explants with DENV and SGE is an alternative method that does not require live mosquitoes and has previously been used to show enhancement of DENV-2 infection in primary keratinocytes [66]. Whether live mosquitoes or SGE is used, there is a substantial gap and opportunity to investigate the role of mosquito saliva on infection in an ex vivo human skin model. 

Although only the infection of explants with DENV was discussed here, a notable benefit of these explant models is the ability to study other arboviruses. Skin explants have been used to investigate the effect of mosquito saliva and TLR7 agonists on ex vivo infection with Semliki Forest virus, cellular tropism of Zika virus, Zika virus transmission by DCs, and ex vivo infection with Mayaro virus [138,139,140,141]. They have also been used to study the effects of sub-neutralizing levels of DENV antibodies during Zika virus infection in an antibody-dependent enhancement model [100]. This work emphasizes the versatility and value of these skin models for studying cutaneous immune responses and antibody-dependent enhancement of infection for a broad range of arboviruses. 

## 7. Closing Remarks and Future Studies

It is estimated that around half of the world’s population is at risk of dengue. Viral transmission and establishment of infection occur in the skin after a mosquito bite, and the early cutaneous immune responses can be the determining factor in whether an infection is controlled or disseminated to the rest of the body. Further studies using human skin cells and explants could help test hypotheses surrounding what causes some patients but not others to progress to severe disease. Host genetic factors and single nucleotide polymorphisms in innate genes, many of which are active in skin immunity, have been associated with dysregulated immune signaling and an increased risk of disease severity [142,143,144,145,146]. Overactivation of the complement cascade in response to primary and secondary DENV infections has also been implicated as a marker for severe dengue [147]. Both host genetic factors and complement activation could be studied using these human skin models to assess their role in immune dysregulation. By investigating the early viral–host interactions in the cutaneous environment, potential targets can be identified for the future development of therapeutics to prevent severe dengue disease. 

## Figures and Tables

**Table 1:** Overview of research using primary human skin cells to study DENV and innate immune responses. Abbreviations: retinoic acid-inducible gene I = RIG-I, melanoma differentiation 5 = MDA5, antimicrobial peptides = AMPs, interferon = IFN, salivary gland extract = SGE, Langerhans cells = LCs, dermal dendritic cells = dDCs, mononuclear phagocytes = MNPs, interleukin = IL, tumor necrosis factor-α = TNF-α, chemokine ligand = CCL, interferon response factor = IRF, antibody-dependent enhancement = ADE. The dark blue signifies the headers and the light blue the body of the table.

Model	Key Takeaways	References
Primary keratinocytes	Keratinocytes are permissive to DENV infection Keratinocytes upregulate RIG-1, MDA5, TLR-3, AMPs, and Type I and III IFNs SGE enhances infection of keratinocytes	Surasombatpattana et al., 2011 [64] Surasombatpattana et al., 2012 [66]
Primary skin mononuclear phagocytes	Keratinocytes are permissive to DENV infection Keratinocytes upregulate RIG-1, MDA5, TLR-3, AMPs, and Type I and III IFNs SGE enhances infection of keratinocytes	Wu et al., 2000 [32] Cerny et al., 2014 [33] Schaeffer et al., 2015 [93] Duyen et al., 2017 [94] Helgers et al., 2023 [95]
Primary fibroblasts	DENV can infect fibroblasts Fibroblasts upregulate RIG-I, IRF-3, and IRF-7 Fibroblasts also upregulate IL-1β, IL-6, IL-8, GM-CSF, TNF-α, and type I and III IFN	Kurane et al., 1992 [34] Diamond et al., 2000 [96] Bustos-Arriaga et al., 2011 [97] Wei et al., 2020 [98] Wei et al., 2023 [99]
Primary cutaneous mast cells	Human skin mast cells are susceptible to primary DENV infection They upregulate CCL5, IL-6, and IL-8 DENV is found in extracellular and cytoplasmic granules These granules are infectious and can disseminate virus to draining lymph nodes	Troupin et al., 2016 [35]
Human skin explants	Immature DCs are more permissive than mature DCs Keratinocytes are a major target of DENV infection IL-1α, IL-1β, IFN-α, and CCL20 are upregulated Cellular recruitment is driven by IL-1β DENV immune sera enhances infection through ADE	Wu et al., 2000 [32] Limon-Flores et al., 2005 [63] Duangkhae et al., 2018 [31] Castanha et al., 2020 [100] Helgers et al., 2023 [95]

## Data Availability

Not applicable.
